# Auricular acupressure for minimizing adverse reactions to colonoscopic bowel preparation in hospitalized patients: A randomized controlled trial

**DOI:** 10.1016/j.heliyon.2025.e42187

**Published:** 2025-01-22

**Authors:** Jiahui Zhang, Chang Liu, Guodong Ruan, Haiyan Zhang, Beiping Zhang, Xuejun Hu, Cailing Zhong

**Affiliations:** a Huangpu Hospital of Guangdong Second Traditional Chinese Medicine Hospital, Guangzhou, Guangdong, 510799, PR China; b Guangdong Provincial Hospital of Chinese Medicine (The Second Affiliated Hospital of Guangzhou University of Traditional Chinese Medicine), Guangzhou, Guangdong, 510120, PR China

**Keywords:** Auricular acupressure, Bowel preparation, Adverse reactions, Vaccaria segetalis, Randomized controlled trial, Traditional Chinese medicine

## Abstract

**Objective:**

To assess the effectiveness and safety of auricular acupressure in reducing the incidence of adverse reactions(ADRs) during the bowel preparation.

**Methods:**

This was a prospective, assessor-blinded, randomized controlled clinical trial implemented at Guangdong Provincial Hospital of Chinese Medicine. Between October 2022 and February 2023, 190 hospitalized patients undergoing colonoscopy were randomly assigned to the intervention and control groups in a 1:1 ratio. The intervention group received auricular acupressure during bowel preparation, whereas the control group received no additional treatment. Analyses were conducted using the intention-to-treat method. Intervention effects were evaluated by comparing outcomes between the two groups.

**Results:**

The overall incidence of ADRs to bowel preparation in the intervention group (37/95 = 37.89 %) was lower than that in the control group (59/95 = 62.11 %, P < 0.05). Compared with control group, the incidence of nausea in the intervention group decreased by 15.79 %(95%CI 0.03–0.19, P = 0.018), whereas no significant difference was observed in the incidence of abdominal distension(P > 0.05). Regarding the comparison of the severity of the ADRs, the overall score of ADRs and the scores for nausea and abdominal distension in the intervention group were statistically lower than those in the control group (all P < 0.05). No auricular acupressure-related adverse effect was observed.

**Conclusions:**

Auricular acupressure can significantly decrease the incidence of ADRs to colonoscopic bowel preparation in patients and alleviate the severity of nausea and bloating symptoms, which is a safe, simple, and effective method.

**Trial registration:**

ChiCTR, no. ChiCTR2200061742; Registered July 2, 2022. URL: https://www.chictr.org.cn/showprojEN.html?proj=167796.

## Highlights

• A TCM trial tested auricular acupressure's effect on adverse reactions to bowel prep.
• Auricular acupressure reduced adverse reactions to bowel prep significantly.
• Nausea and abdominal distension severity were reduced by auricular acupressure.
• Auricular acupressure may offer a new treatment for adverse reactions to bowel prep.

## Graphical abstract

**Figure:**
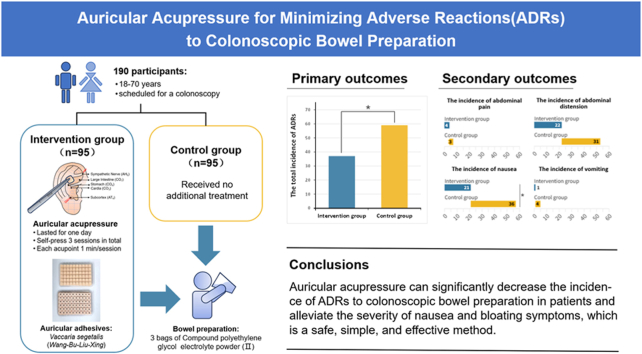

## 1 Introduction

Colonoscopy is a commonly used clinical method for the diagnosis and treatment of colorectal diseases. High-quality colonoscopy plays a crucial role in determining the diagnostic and therapeutic effacts of colorectal diseases [1], particularly in colorectal cancer screening and early diagnosis and treatment [2,3]. The quality of a colonoscopy is closely related to the quality of bowel preparation, and adequate bowel preparation directly impacts the accuracy of the colonoscopy and the safety of the treatment [[4], [5], [6]]. Previous studies have shown that a lower quality of bowel preparation leads to a defective examination field of view, increasing the risk of missed detection of colorectal lesions [7]. This not only increases the rate of repeated colonoscopy and healthcare costs, but also raises the risk of developing interstitial colorectal cancer [8,9].

Adverse reactions (ADRs) to laxatives can significantly impact the effectiveness of bowel preparation. In various bowel preparation regimens, patients are instructed to consume a minimum of 2–4 L of intestinal cleansers, such as polyethylene glycol (PEG), within a short timeframe. This process carries an approximate 42%–59.3 % probability of resulting in gastrointestinal ADRs like nausea, vomiting, and bloating [10,11]. Although these conditions do not result in serious adverse events or compromise patient safety, they can reduce patient adherence to medication and thus affect the quality of bowel preparation [12]. Hence, it is crucial to alleviate the ADRs associated with oral intestinal cleansers, enabling patients to undergo colonoscopic bowel preparation in a safe and comfortable manner. Split dosing, development of appropriate laxative dosages, application of expectorants, and improvement of the taste of intestinal cleansers can, to a certain extent, enhance patients' adherence to medication and alleviate nausea, bloating, and other discomforts, but all of them are ineffective and the efficacy of the interventions is uncertain. Selection of appropriate laxative doses, application of expectorants, and modification of the taste of intestinal cleansers can, to some extent, improve patients' medication compliance and reduce nausea, bloating, and other discomforts. However, these measures still have unsatisfactory effects and uncertain intervention efficacy [[13], [14], [15]].

Auricular acupressure, a well-established and non-invasive strategy in traditional Chinese medicine (TCM), is commonly utilized to relieve various digestive discomfort symptoms. By pressing on the ear points, it can promote the release of neurotransmitters and influence neurohumoral regulation, which together regulate the organs and endocrine functions [[16], [17], [18]]. Studies have shown that auricular acupressure can promote intestinal peristalsis and relieve the symptoms associated with functional constipation, and is also effective in treating chemotherapy-induced nausea and vomiting [17,[19], [20], [21]]. Auricular acupoint application is recommended by Chinese expert guidance as an adjunctive intervention to prevent symptoms such as pain, nausea, and vomiting during the perioperative period [22,23]. To date, no high-quality studies have been conducted on the use of auricular acupressure for ADRs during colonoscopic bowel preparation. As a result, this study aimed to explore whether auricular acupressure could reduce the incidence of ADRs associated with colonoscopic bowel preparation, and to provide an evidence-based basis for the establishment of an intervention program for ADRs to colonoscopic bowel preparation with TCM characteristics.

## 2 Methods

### 2.1 Study design

This prospective, assessor-blinded, randomized controlled clinical trial was conducted at Guangdong Provincial Hospital of Chinese Medicine in China. Eligible inpatients who underwent a colonoscopy were randomly assigned to the intervention and control groups in a 1:1 ratio. The intervention group received auricular acupressure during bowel preparation, while the control group received no additional treatment. Intestinal intolerance was evaluated immediately after bowel preparation. This trial was designed and reported in accordance with the Consolidated Standards of Reporting Trials (CONSORT) [24] and the Standards for Reporting Interventions in Clinical Trials of Acupuncture (STRICTA) [25] guidelines.

### 2.2 Participants

#### 2.2.1 Inclusion criteria

The inclusion criteria were as follows: (1) patients scheduled for a colonoscopy at our institution; (2) males or females aged between 18 and 70 years; (3) patients who were willing to participate in the trial and had signed the informed consent form.

#### 2.2.2 Exclusion criteria

The exclusion criteria were (1) patients with severe mental disorders; (2) patients with serious intestinal diseases; (3) patients with contraindications to colonoscopy; (4) patients with severe liver, kidney, heart, brain, lung dysfunction, or neurological diseases; (5) patients who had intolerance to colonoscopy; (6) patients with menstrual cycle, pregnancy, or breastfeeding; (7) patients with allergies to drugs and other ingredients used in the trial; (8) patients who had skin lesions at the to-be-treated auricular acupoints or allergy to ear adhesive.

#### 2.2.3 Recruitment and withdrawal

Eligible inpatients who satisfied the inclusion criteria were recruited from Guangdong Provincial Hospital of Chinese Medicine. All admitted patients who met the initial eligibility criteria were asked if they would like to participate. Participants were permitted to withdraw from the trial at any time for any reason.

### 2.3 Sample size

According to the data from our pilot study, the incidence of ADRs to bowel preparation was expected to be 60 % in the control group and 34 % in the intervention group. In accordance with the 1:1 parallel control principle, the PASS statistical analysis software package was utilized to compute the sample size for a two-sided significance level of 0.05 and a power of 90 % (α = 0.05, β = 0.1). Considering a 20 % dropout rate of patients in the trial, 190 patients were enrolled. (n = 95 in each group).

### 2.4 Randomization and blinding

Preassigned assistant researchers were responsible for the randomization process. Excel was used to generate 190 random numbers within the range of 0–1. These numbers were then sequenced in descending order, with the largest random number being assigned the value of 1 and the smallest being 190. Subsequently, the sequenced numbers were matched with the random numbers and placed on individual cards, which were then placed in a sealed opaque envelope. Following the acquisition of informed consent from the participants and the completion of baseline data collection, the assistant researchers drew cards from the envelope. Participants with odd sequence numbers were allocated to the control group, while those with even sequence numbers were assigned to the auricular acupuncture group. The acupuncturists were then informed of the group assignments.

This study was designed as an assessor-blinded clinical trial due to the unique features of the intervention. Before data collection, subjects in the intervention group were instructed to remove the auricular adhesives, which was checked by the personnel in charge of randomization. Throughout the study period, the participants and other researchers were prohibited from revealing the groupings to the endoscopists, data processors, or statisticians. To avoid communication between the two groups, the patients were sent to separate wards.

### 2.5 Auricular acupressure materials

**Fig. 1:**
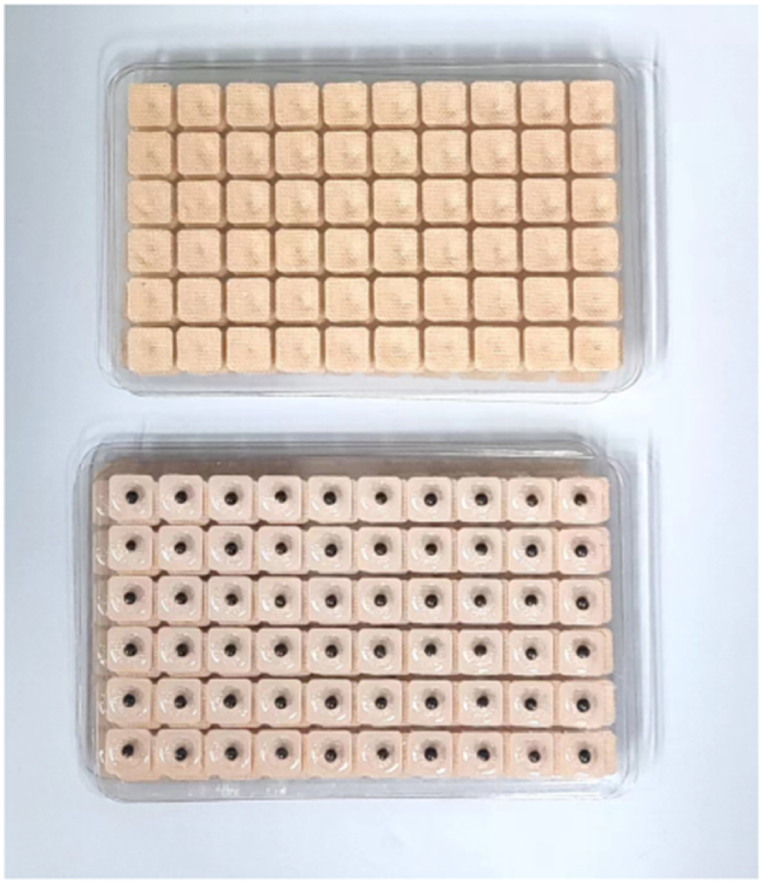
Auricular adhesives.

The auricular adhesives (Hebei Heshi Medical Instrument Co., Ltd., China), measuring 6 mm × 6 mm in size, with a 2 mm diameter *Vaccaria segetalis* (VS) (the dried and mature seeds of *Vaccaria segetalis* (Neck.) Garcke ex Asch; *Wang-Bu-Liu-Xing* in Chinese) in the center, were used in the experiment (see Fig. 1).

### 2.6 Intervention

#### 2.6.1 Routine bowel preparation scheme

All participants were limited to consume only low-residue foods the day before the colonoscopy. All subjects underwent identical bowel preparation and received 3 bags of Compound PEG electrolyte powder (Ⅱ) (Shenzhen Wanhe Pharmaceutical Co. Ltd., China, 68.56 g/bag, H20030827), each containing 1.46 g of sodium chloride, 5.68 g of anhydrous sodium sulfate, 0.74 g of potassium chloride, 1.68 g of sodium bicarbonate, and 59 g of PEG 4000. Each PEG sachet was diluted in 1 L of drinking water and consumed within 1 h. Subjects scheduled for two different time slots (morning or afternoon) were instructed to drink 1 L of laxative solution at 8:00 p.m. before the colonoscopy. Subjects undergoing colonoscopy in the morning drunk the remaining 2 L from 4:00 to 6:00 a.m. on the day of the examination, whereas subjects undergoing colonoscopy in the afternoon drunk from 10:00 to 12:00 a.m. A bottle of simethicon (Berlin-Chemie AG, Germany; 30 ml/bottle, HJ20160184) was added to the last 1 L of the laxative. When taking laxatives, the participants were instructed to move around and massage the abdomen clockwise to facilitate bowel movements.

#### 2.6.2 Intervention group

**Fig. 2:**
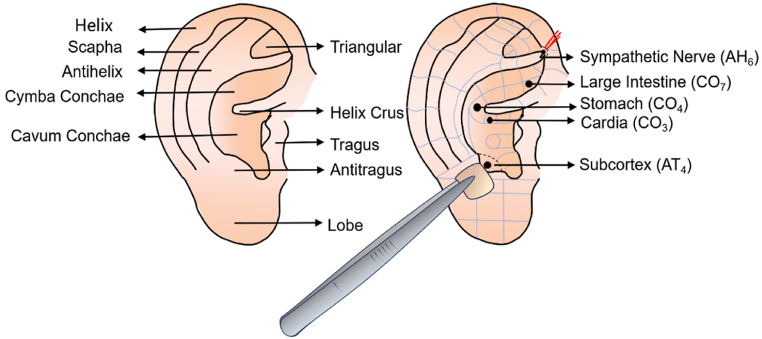
Anatomical locations of the auricular acupoints.

Subjects in the intervention group received auricular acupressure administered by one of four acupuncturists with over three years of treatment experience. In the intervention group, auricular adhesives were applied to particular spots in both ears for one day, starting one day before colonoscopy and continuing until the completion of bowel preparation. According to the clinical experience of our research group and the effective prescription published previously [19,26], five acupoints (Cardia (CO3), Stomach (CO4), Large Intestine (CO7), Sympathetic Nerve (AH6), and Subcortex (AT4)) were selected as the auricular acupoint protocols in this study. The nomenclature and location of auricular acupoints we adopted were stipulated by the World Federation of Acupuncture-Moxibustion Societies (WFAS) in 2013 [27]. The anatomical locations are listed in Table 1 and illustrated in Fig. 2. Since subjects were instructed to self-press the adhesives three times while drinking the laxative solution. Each acupoint would be pressed for at least 1 min per session, and the pressure would elicit a distending and aching sensation within the subject's tolerance. Medical staff promptly reminded and instructed the patient about the timing of the laxative. If skin abrasion or swelling occurred at the treated site, the subject need to contact the acupuncturist immediately to solve it, which would be recorded in a case report form (CRF).

**Table 1:** Location of the auricular acupoints.

Acupoint	Location
Cardia (CO_3_)	In the concha inferior to the posterior 1/3rd of the helix crus.
Stomach (CO_4_)	At the end of the helix crus.
Large Intestine (CO_7_)	At the anterior 1/3rd of the region between the helix crus and Line AB.
Sympathetic Nerve (AH_6_)	At the juncture of the end of the inferior antihelix crus and the medial edge of the helix.
Subcortex (AT_4_)	On the medial side of the antitragus.

#### 2.6.3 Control group

The subjects in the control group only received a routine bowel preparation scheme without any supplementary means for ADR prevention.

#### 2.6.4 Colonoscopy procedures

All the colonoscopies were performed by an experienced endoscopist. Colon cleanliness was assessed by the endoscopist and two experienced endoscopy nurses who were not involved in the trial. Three researchers were blinded to the intervention allocation and independently judged whether each colon had no staining, minor staining, or residual stool after the operation. The research nurse registered the prevailing consensus.

### 2.7 Outcome measures

The primary outcome measure was the total incidence of ADRs to colonoscopic bowel preparation, including the incidence of abdominal pain, abdominal distension, nausea, and vomiting after PEG intake. The secondary outcome measures were (1) incidence of each adverse event; (2) overall score of ADRs and corresponding score of each adverse event; (3) qualified rate of bowel preparation: assessed using the Boston Bowel Preparation Scale (BBPS), with a qualified rate defined as the ratio of subjects with a BBPS score of >6 [28]; (4) 100 % PEG solution intake rate: whether the patient took the complete laxative as ordered (yes or no); (5) procedure time of drinking PEG: defined as the time spent drinking PEG (in minutes); (6) the rate of taking additional PEG; (7) patients' willingness to undergo colonoscopy in the future (yes or no). In addition, the Likert scoring method was used to assess the severity of abdominal pain, abdominal distension, and nausea symptoms. For this scale, 0 indicates no discomfort, while 10 indicates an inability to tolerate due to strong discomfort. Vomiting severity was assessed based on frequency. If the subject vomited once, it was recorded as 2 points; if the subject vomited more than or equal to 4 times, it was recorded as 10 points.

### 2.8 Safety assessment

Allergic reactions such as skin flushing, rash, exudation, and ulceration may occur during the treatment evaluation period. If an allergic reaction occurred, auricular acupressure on the ear should be stopped immediately. Adverse effects related to auricular acupressure should be documented and addressed promptly.

### 2.9 Data collection

All baseline data for each participant were collected before the intervention. Baseline data included sex, age, body mass index (BMI), past medical history, medication history, recent defecation, indications for colonoscopy, and gastrointestinal disorders before bowel preparation, and so on. To facilitate researchers in observing and recording various indicators, the information of each subject was documented in the CRF using codes and abbreviations instead of names to ensure privacy. The data in the CRF form was independently entered and verified by two third-party data managers who were not affiliated with the test to confirm its authenticity, accuracy, and integrity. The participants' medical records (including study medical records, CRF, colonoscopy results, etc.) were stored at the hospital. No public report of the results of this study would reveal the personal identities of the participants.

### 2.10 Quality control

To ensure the quality of this study, the trial underwent several changes and revisions by relevant digestive disease specialists, acupuncture experts, professional statisticians, and research methodologists. Strict inclusion and exclusion criteria were established. To maintain objectivity, we ensured that the data collection statisticians and endoscopy operators were blinded. All the researchers, especially the auricular acupressure operators, had completed our training, ensuring consistent treatment practices and uniformity in the terminology used for communication with the subjects. The CRFs were meticulously completed in accordance with the CRF guidelines and checked by third-party data administrators unrelated to the trial to ensure the scientific accuracy of the data. When suspicious data was found, the researcher would be contacted and verified, and the data would then be corrected, validated, and entered by the data manager based on the researcher's response. All quality control documents were completed and filed as required.

### 2.11 Statistical analysis

IBM SPSS (version 25.0, IBM, Armonk, NY, USA) was used for data processing. The measurement data was expressed as means ± standard deviation or median (interquartile range), and the counting data was described by the number of cases (percentage). The data was analyzed using the principle of intention-to-treat. That was, regardless of whether the subjects received the treatment of the assigned group, they would be included in the allocated group for the statistical analysis of the curative effect. Efficacy and safety measures were analyzed in accordance with “per-protocol”, whereby we included all subjects who completed the entire trial and followed the protocol requirements. Fisher's exact test or Pearson's chi-square test was used for trial group comparisons of the incidence of ADRs. Differences in quantitative data such as age, BMI, the scores of various adverse events, and procedure time of drinking PEG were compared between the experimental and control groups by using the *t*-test for comparison of two samples and the rank-sum test for non-normal distribution or unevenness of variance. Two-tailed tests with P < 0.05 were defined as statistically significant for all analyses.

### 2.12 Patient and public involvement

The patients were not involved in the design or conduct of the study. However, patients who exhibited symptoms of intolerance to bowel preparation in our clinical department were consulted before the trial design. The auricular acupoints, treatment frequency, and trial duration were summarized based on our clinical experience and patient feedback.

### 2.13 Ethics statement

This study was approved by the Institutional Ethics Committee of Guangdong Provincial Hospital of Chinese Medicine on May 1, 2022 (approval no. YF2022-093). Trial registration: chictr. org.cn; no. ChiCTR2200061742 (https://www.chictr.org.cn/showprojEN.html?proj=167796). The experimental design and informed consent forms were developed in compliance with the Declaration of Helsinki (as revised in 2013) [29]. All qualified participants were informed of the study details and required to sign written informed consent forms before participating. During the study and data collection process, no ethical concerns arose. Participants were guaranteed confidentiality of their information, and all data was analyzed anonymously.

## 3 Results

### 3.1 Patients

**Fig. 3:**
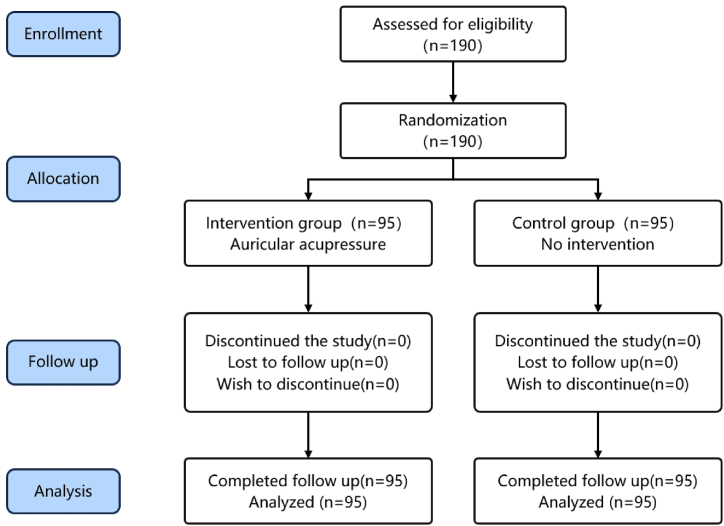
Study flow diagram.

Fig. 3 shows that 190 hospitalized patients undergoing colonoscopy were enrolled and randomized between October 2022 and February 2023, with 95 assigned to the intervention group and 95 to the control group. No cases were excluded or discontinued in either group, and a total of 190 cases successfully completed the clinical trial.

General baseline data on the enrolled patients was collected. The chi-square test or Fisher exact test results revealed that the observed differences between the two groups in gender, BMI, hypertension history, diabetes history, abdominal surgery history, family history of colorectal cancer, the use of tricyclic antidepressants, smoking history, drinking history, the use of tricyclic antidepressants, the proportion of type 1 or type 2 in the Bristol stool chart, the number of previous colonoscopy examinations, the purpose of this examination, the symptoms before the examination, and the colonoscopy anesthesia status were not statistically significant. The normality test for age in both groups yielded a result of P < 0.05, indicating a lack of normality. As a result, the data was presented using the median and interquartile range (M (P25, P75)), and the comparison of the two groups was performed using the Mann-Whitney *U* test, showing no statistically significant difference in age between the two groups (P > 0.05). Overall, the baseline of patients in both groups was consistent and comparable (see Table 2).

**Table 2:** Baseline characteristics of enrolled patients.

Items	Intervention group(n = 95)	Control group(n = 95)	*χ*^2^*/t/Z*	P value
**Gender (Female), n (%)**	44 (46.3 %)	42 (44.2 %)	0.085	0.771^a^
**Age(years), M (P25, P75)**	58 (48, 64)	60 (52, 65)	1.120	0.263^c^
**BMI (kg/m**^**2**^**), n (%)**				
≧24	35 (36.80 %)	44 (46.30 %)	1.860	0.394^a^
<18.5	7 (7.40 %)	7 (7.40 %)		
**Anamnesis, n (%)**				
Hypertension	24 (25.30 %)	23 (24.20 %)	0.028	0.866^a^
Diabetes	10 (10.53 %)	11 (11.53 %)	0.054	0.817^a^
Abdominal surgery	17 (17.90 %)	13 (13.70 %)	0.633	0.426^a^
Family history of colorectal cancer	6 (6.30 %)	5 (5.30 %)	0.096	0.756^a^
The use of tricyclic antidepressants	0 (0)	0 (0)	–	1.000
**Habits, n (%)**				
Smoking	19 (20 %)	19 (20 %)	–	1.000
Drinking	14 (14.70 %)	13 (13.70 %)	0.043	0.835^a^
**Bowel movements, n (%)**				
The frequency of stool <3 times per week	3 (3.2 %)	3 (3.2 %)	–	1.000
Type 1 or 2 in Bristol stool chart	10 (10.50 %)	14 (14.70 %)	0.763	0.382^a^
**Number of previous colonoscopy examinations, n (%)**				
0 times	11(11.60 %)	6(6.30 %)	1.754	0.416^a^
1-2 times	72(75.80 %)	78(82.10 %)		
≥3 times	12(12.60 %)	11(11.60 %)		
**Purpose of this examination, n (%)**				
Health screening	9(9.50 %)	6(6.30 %)	1.103	0.573^b^
Disease diagnosis/treatment	84(88.40 %)	88(92.60 %)		
Disease follow-up	2(2.10 %)	1(1.05 %)		
**Symptoms before the examination and the colonoscopy anesthesia status, n (%)**				
Abdominal pain, abdominal distension, nausea and vomiting before examination	18(18.90 %)	24(25.30 %)	1.100	0.294^a^
General anesthesia	95(100 %)	95(100 %)	–	1.000

Abbreviations: BMI: Body mass index.

a P value compared using chi-square test.

b P value compared using Fisher exact test.

c P value compared using Mann-Whitney *U* test.

### 3.2 Primary outcome

The overall incidence of ADRs to bowel preparation in the intervention group (37/95 = 37.89 %) was lower than that in the control group (59/95 = 62.11 %, P < 0.05, see Table 3).

**Table 3:** Comparison of the total incidence of ADRs to bowel preparation between two groups.

	Number(n)	Incidence of ADRs, n (%)	No appearance of ADRs, n (%)	*χ*^*2*^	P value
Intervention group	95	37(38.95 %)	58(61.05 %)	10.191	0.001^a^^,^^b^
Control group	95	59(62.11 %)	36(37.89 %)		

a Denotes P value significant at 5 % level of significance.

b P value compared using chi-square test.

### 3.3 Secondary outcomes

**Fig. 4:**
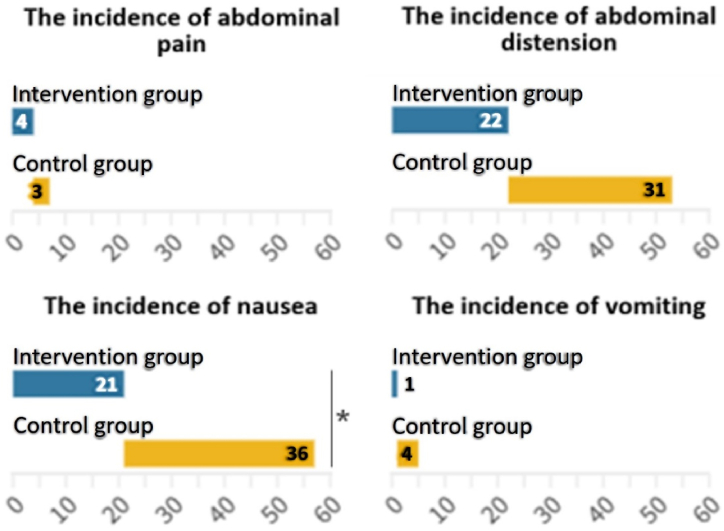
Incidence of each adverse event.

**Fig. 5:**
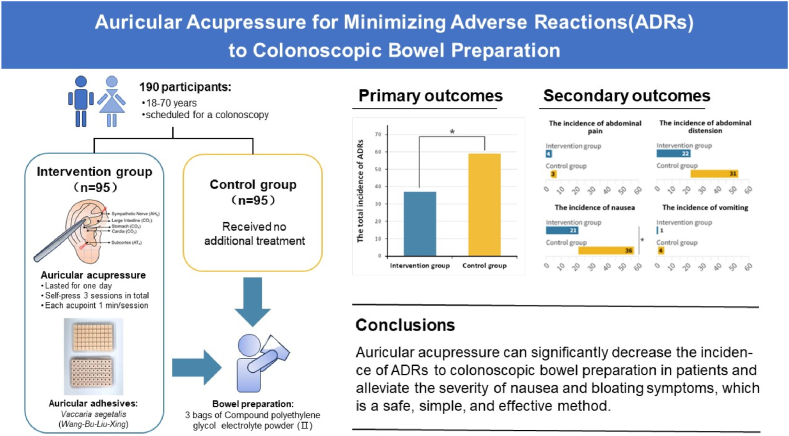
Graphical abstract.

Compared with control group, the incidence of nausea in the intervention group decreased by 15.79 %(95%CI 0.03–0.19, P = 0.018), whereas no significant difference was observed in the incidence of abdominal pain, abdominal distension, and vomiting (all P > 0.05). Abdominal distension and nausea were the most prevalent adverse events in the two groups, comprising over 90 % of all occurrences (see Table 4 and Fig. 4) (see Fig. 5).

**Table 4:** Comparison of secondary outcomes between groups.

	Intervention group(n = 95)	Control group(n = 95)	*χ*^2^/*t*/*Z*	P value
**Incidence of each adverse event, n (%)**				
Abdominal pain	4(4.21 %)	3(3.16 %)	–	1.000^b^
Abdominal distension	22(23.22 %)	31(28.42 %)	2.120	0.145^c^
Nausea	21(22.10 %)	36(37.89 %)	5.639	0.018^a^,^c^
Vomiting	1(1.10 %)	4(4.21 %)	0.822	0.365^c^
**Overall score of ADRs and corresponding score of each adverse event, M (P25, P75)**				
Total ADRs	0(0, 2)	2(0, 5)	3.073	0.002^a^,^d^
Abdominal pain	0(0, 0)	0(0, 0)	0.326	0.744^d^
Abdominal distension	0(0, 0)	0(0, 3)	2.08	0.038^a^^,^^d^
Nausea	0(0, 0)	0(0, 3)	2.702	0.007^a^^,^^d^
Vomiting	0(0, 0)	0(0, 0)	1.361	0.174^d^
**Qualified rate of bowel preparation, n (%)**				
Qualified	91(95.80 %)	78(91.60 %)	1.423	0.233^b^
Unqualified	4(4.20 %)	8(8.40 %)		
**BBPS score [M(P25, P75)]**				
Total score	7(7–8)	7(7–7)	1.286	0.199^d^
Left hemicolon	2(2–2)	2(2–2)	1.626	0.104^d^
Transverse colon	3(3–3)	2(2–3)	1.152	0.249^d^
Right hemicolon	2(2–3)	2(2–2.5)	0.751	0.453^d^
**Others**				
100 % PEG solution intake(Yes), n (%)	92(96.8 %)	92(96.8 %)	–	1.000
Procedure time of drinking PEG(min), M(P25, P75)	98.23 (98, 120)	91.26 (80, 90)	1.294	0.296^d^
The rate of taking additional PEG, n(%)	1(1.1 %)	2(2.1 %)	0.000	1.000^c^
Patients' willingness to undergo colonoscopy in the future, n(%)	95(100 %)	95(100 %)	–	1.000

Abbreviations: ADRs: Adverse reactions; BBPS: The Boston Bowel Preparation Scale; PEG: Polyethylene glycol.

a Denotes P value significant at 5 % level of significance.

b P value compared using Fisher exact test.

c P value compared using chi-square test.

d P value compared using Mann-Whitney *U* test.

Regarding the comparison of the severity of the ADRs, the overall score of ADRs and the score for nausea and abdominal distension in the intervention group were statistically lower than those in the control group (P < 0.05; see Table 4).

Qualified rate of bowel preparation in the intervention group was higher than that in the control group (P < 0.05), whereas no significant difference was found when it came to Boston Bowel Preparation Score (P > 0.05; see Table 4).

100 % PEG solution intake rate, procedure time of drinking PEG, the rate of taking additional PEG, and patients' willingness to undergo colonoscopy in the future did not differ statistically between the two groups (all P > 0.05; see Table 4).

### 3.4 Adverse effects

The intervention group experienced no auricular acupressure-related adverse effect.

## 4 Discussion

This study first demonstrated that the incidence of ADRs during bowel preparation was significantly lower in the auricular acupressure group compared to the blank control group, with a statistically significant difference (P < 0.05). Bloating and nausea, accounting for more than 90 % of all ADRs, were among the most common. Further analysis revealed that auricular acupressure resulted in a lower incidence of abdominal distension compared to the control group, although the difference was not statistically significant (P > 0.05). However, the incidence of nausea was 15.79 % lower in the auricular pressure group compared to the control group (95 % CI 0.03–0.19, P = 0.018). These findings suggest that auricular acupressure may reduce the overall incidence of ADRs to bowel preparations by decreasing the likelihood of nausea (P < 0.05).

When evaluating the severity of ADRs between the two groups, it was noted that the auricular acupressure group exhibited lower total adverse reaction scores, as well as scores for nausea and abdominal distension events, in comparison to the blank control group, with statistically significant differences. This indicates that auricular acupressure can alleviate the severity of ADRs during bowel preparation, particularly in reducing bloating and nausea.

Auricular acupressure is a practical TCM external treatment method that stimulates the skin of acupoints by means of small spherical magnetic pills or seeds. In this trial, auricular adhesives made of VS were used for the intervention. With its firm texture and appropriate size, VS can be easily applied to auricular points, providing non-penetrating pressure stimulation to specific areas for extended periods without the risk of ADRs such as infections associated with penetrating needles, making it an effective and safe option for long-term use in auricular acupressure [30]. TCM posits that sustained stimulation through auricular acupressure is effective in benefiting qi and promoting blood circulation to remove obstructions in the collaterals, regulating the functions of the corresponding meridians and viscera [21,31,32]. Additionally, modern studies have proven that VS has anti-inflammatory effects, preventing inflammation caused by the pressure of the seeds on the ear or the irritating effect on the skin, and is capable of applying precise pressure on the specific acupoints [21,33]. Inspired by auricular acupressure, transcutaneous auricular vagus nerve stimulation (taVNS) is a non-invasive therapeutic technique developed by the China Academy of Chinese Medical Sciences. It has been implemented in scientific research and clinical settings [34,35]. Previous studies have confirmed that auricular acupressure and taVNS can regulate the autonomic nervous system through vagal mediation, further modulating gastrointestinal dynamics [[36], [37], [38]]. The stimulus signals originating from the auricle are likely transmitted by the vagus nerve to the nucleus of the solitary tract, which then stimulates the autonomic centers of the cerebral cortex and projects to other areas of the brain [16]. Conversely, the dorsal motor nucleus of the vagus nerve transmits signals downward to the stomach and other peripheral organs, thereby enhancing gastric emptying [39]. Concurrently, auricular acupoint therapy modulates gastrointestinal hormones and is significant in maintaining the balance of the internal environment. Previous studies have found that motilin (MOT) levels were significantly increased in patients after gynecologic laparoscopy under general anesthesia, and supraphysiologic doses of MOT could cause strong temporal contractions of the entire small intestine, leading to gastrointestinal smooth muscle spasms, nausea, vomiting, and other discomforts [40,41]. A study [41] suggested that auricular acupressure could reduce the incidence of postoperative nausea and vomiting in patients and improve gastrointestinal function by inhibiting the excessive release of plasma MOT. Overall, auricular acupressure has a comprehensive impact on neural-fluid regulation to expedite the restoration and maintenance of normal gastrointestinal function. Auricular acupressure may be the mechanism by which the discomfort degree of abdominal distension and nausea during bowel preparation is reduced, as well as the incidence of nausea.

The auricular points selected for this study were Cardia (CO3), Stomach (CO4), Large Intestine (CO7), Sympathetic Nerve (AH6), and Subcortex (AT4). Studies have shown that Stomach (CO4) and Large Intestine (CO7) are primarily utilized to address postoperative abdominal distension after uterine fibroid surgery [40,42]. Meanwhile, Stomach (CO4) and Cardia (CO3) are most commonly chosen for treating postoperative nausea and vomiting after breast cancer, and they have a palliative effect on the symptoms of gynecologic postoperative laparoscopy [43]. Clinical practice has also observed that Cardia (CO3) serves to harmonize the stomach and stop vomiting, making it a key point for addressing vomiting and nausea. Subcortex (AT4) can regulate the function of the digestive system and relieve nausea and vomiting caused by nerve reflexes [44]. Large intestine (CO7) can stimulate intestinal mobilization, facilitate defecation, and alleviate abdominal distension [45,46]. As demonstrated [41], the combination of auricular acupoints for Cardia (CO3), Stomach (CO4), Sympathetic Nerve (AH6), and Subcortex (AT4) can help improve gastrointestinal function by stimulating the vagus nerve, regulating autonomic nerves, and inhibiting excessive release of plasma MOT. For these reasons, the recommended acupoints for auricular acupressure to reduce ADRs of colonoscopic bowel preparation were selected in this study as Cardia (CO3), Stomach (CO4), Large Intestine (CO7), Sympathetic Nerve (AH6), and Subcortex (AT4).

Previous studies have also found that auricular acupressure in reducing the occurrence of ADRs to colonoscopic bowel preparation. For instance, Wang et al. [47] conducted a study where auricular acupressure was administered to 90 patients undergoing colonoscopy, resulting in a reduction of perianal discomfort, nausea, and vomiting during bowel preparation. Similarly, Xing [48] investigated 100 patients and observed that auricular acupressure decreased ADRs such as nausea, abdominal distension, and abdominal pain associated with bowel preparation. Despite the significant findings in these studies, the sample sizes were relatively small. Additionally, Xing's study [48] applied auricular acupressure to the experimental group three days before the procedure, while Wang's study [47] administered it one day prior, involving 3 to 5 presses lasting 1–2 min each. Although the shorter intervention duration in this study compared to the aforementioned studies, similar positive outcomes were observed, indicating the necessity for further research to establish the optimal auricular acupressure intervention protocol.

Inadequate bowel preparation has the most prominent impact on the accuracy of colonoscopy, in addition to an elevated risk of complications such as bleeding and perforation [49,50]. The evaluation scale used in this study to assess the quality of bowel preparation was BBPS, which is also an internationally recognized evaluation scale [51]. However, the comparison of total BBPS scores and the scores of each bowel segment in the two groups did not show statistical significance (P < 0.05). This indicates that auricular acupressure did not lead to an improvement in the quality of bowel preparation among patients, contrary to findings reported in earlier studies [47,48,52]. It might be related to the fact that the wards have strict quality control sessions for bowel preparation. Additional laxative doses will be administered to hospitalized patients with poor bowel cleansing, so bowel cleansing is not likely to differ between the two groups. Other factors that may have contributed to the results include a small sample size, an insufficient duration of intervention, and inadequate frequency of action. Studies with larger samples are necessary to further investigate the effects of auricular acupressure on BBPS.

Despite the positive results of our experiments, we must recognize that our study has limitations. Firstly, double-blinding could not be implemented. The absence of a blank control group for routine bowel preparation instruction intervention and the lack of a sham auricular acupressure intervention to counteract the placebo effect resulted in a failure to fully and adequately validate the effectiveness of auricular acupressure in reducing the incidence of colonoscopic bowel preparation ADRs. Auricular diagnostic methods that investigate auricular points unrelated to the regulation of gastrointestinal function, serving as a control group, through auricular potential characterization measurements may help mitigate the impact of psychological or physiological reactions of the study participants on the effectiveness of the intervention. Second, the stimulation of the auricular points involved applying pressure to the auricular adhesives three times while drinking laxative solution, with each application lasting at least 1 min at each acupoint. However, it did not allow for a comprehensive assessment of the frequency at which auricular acupressure points are effective for all patients. Finally, this study was conducted in a single center with a small sample size, which limits the generalizability of our findings. More high-level clinical trials are needed in the future to further validate the evidence-based basis for the occurrence of ADRs to auricular acupressure interventions.

## 5 Conclusions

This study demonstrated that auricular acupressure can significantly decrease the incidence of ADRs to colonoscopic bowel preparation in patients and alleviate the severity of nausea and bloating symptoms, providing a new clinical intervention to mitigate the ADRs associated with colonoscopic bowel preparation. Auricular acupressure is a safe, simple, and effective method with promising potential for reducing ADRs and improving the tolerability of colonoscopic bowel preparation in patients. It is worth further in-depth study.

## CRediT authorship contribution statement

**Jiahui Zhang:** Writing – original draft, Formal analysis, Conceptualization. **Chang Liu:** Writing – original draft, Visualization, Formal analysis. **Guodong Ruan:** Writing – review & editing, Data curation. **Haiyan Zhang:** Visualization, Methodology. **Beiping Zhang:** Resources, Project administration. **Xuejun Hu:** Supervision, Project administration, Conceptualization. **Cailing Zhong:** Writing – review & editing, Validation, Supervision.

## Availability of data and materials

The datasets generated and/or analyzed during the current study areavailable from the corresponding author on reasonable request.

## Ethics approval and consent to participate

All participants provided written informed consent and the ethical approval was granted on May 1, 2022 by the Ethics Committee of Guangdong Provincial Hospital of Chinese Medicine (approval No. YF2022-093).

## Consent for publication

Not applicable.

## Funding

This trial is supported by the Administration of Traditional Chinese Medicine of Guangdong Province, China (20232054), the Guangdong Provincial Key laboratory of Chinese Medicine for Prevention and Treatment of Refractory Chronic Diseases, Guangzhou Municipal Department of Science and Technology 2025 Municipal School (Academy) Enterprise Joint Funding Special Project (Grant number SL2024A03J00115), the University Research Project of 10.13039/501100010226Guangdong Provincial Department of Education (Grant number 2021ZDZX2059), and the "Double First-Class" and High-Level University Discipline Collaborative Innovation Team of 10.13039/501100010618Guangzhou University of Chinese Medicine (Grant number 2021xk58).

## Declaration of competing interest

The authors declare that they have no known competing financial interests or personal relationships that could have appeared to influence the work reported in this paper.

## Abbreviations:

**PEG**: Polyethylene glycol
**ADRs**: Adverse reactions
**TCM**: Traditional Chinese Medicine
**CONSORT**: The Consolidated Standards of Reporting Trials
**STRICTA**: The Standards for Reporting Interventions in Clinical Trials of Acupuncture
**VS**: *Vaccaria segetalis*
**CRF**: Case report form
**BBPS**: The Boston Bowel Preparation Scale
**BMI**: Body mass index
**taVNS**: Transcutaneous auricular vagus nerve stimulation
**MOT**: Motilin
