# Supplementary material for: Auricular acupressure for minimizing adverse reactions to colonoscopic bowel preparation in hospitalized patients: A randomized controlled trial
Source: Heliyon. 2025 Jan 22;11(3):e42187. doi: 10.1016/j.heliyon.2025.e42187 (PMC11848087; doi:10.1016/j.heliyon.2025.e42187)
Supplement: Multimedia component 1 [file mmc1.docx]

Auricular acupressure for adverse reactions to colonoscopic bowel preparation:

a randomized controlled trial protocol

**Case Report Form**

(Translated from Chinese)

Participant ID：

Name abbreviation of Participant ：

Name abbreviation of investigator：

Date of recruitment：__ __ (Day) __ __ (Month) __ __ __ __ (Year)

**Selection criteria**

| Inclusion criteria | Yes | No |
| --- | --- | --- |
| 1. In-patients will anticipate a colonoscopy at our institution | 🞎 | 🞎 |
| 1. Ages 18 to 70 years old | 🞎 | 🞎 |
| 1. Signed informed consent | 🞎 | 🞎 |

If any of the above items is answered with “No”, the patient can not be recruited into the trial.

| Exclusion criteria | Yes | No |
| --- | --- | --- |
| 1. Severe mental disorder | 🞎 | 🞎 |
| 1. Serious intestinal diseases, such as such as intestinal lumen stenosis, obstruction, intestinal bleeding | 🞎 | 🞎 |
| 1. Contraindications to colonoscopy, such as severe liver, kidney, heart, brain, lung dysfunction or neurological diseases | 🞎 | 🞎 |
| 1. Patients intolerant of colonoscopy | 🞎 | 🞎 |
| 1. Patients in their menstrual cycle, pregnancy or breastfeeding | 🞎 | 🞎 |
| 1. Patients allergic to the drugs and other ingredients used in the trial | 🞎 | 🞎 |
| 1. Skin lesions such as burns, eczema, ulcers, frostbite ulceration and other contraindications at the to-be-treated auricular acupoints or allergy to the ear adhesive. | 🞎 | 🞎 |

If any of the above items is answered with “Yes”, the patient could not be recruited in the trial.

| Informed consent |
| --- |

Date of signing informed consent: __ __ (Day) __ __ (Month) __ __ __ __ (Year)

(Attach Allocation Card Here)

**Baseline assessment**

1. General information

1.1. Age: 🞎🞎

1.2. Gender: 🞎Male 🞎Female

1.3. Nationality: 🞎Han 🞎Other: __________

1.4. Height: 🞎🞎🞎cm Weight: 🞎🞎🞎kg

1.5. Degree of education: 🞎 no formal education 🞎Primary school 🞎Junior high school 🞎Senior high school or

technical secondary school 🞎Junior college or bachelor degree 🞎Master degree or above

1.7. Occupation: 🞎Worker 🞎Farmer 🞎Student 🞎 Politician 🞎Professional 🞎Retired 🞎 Freelancer 🞎Other: ________

1. Past medical history

🞎No 🞎Yes (If yes, please record the following details)

2.1. History of hypertension: years, the most commonly used drugs:

2.2. History of diabetes: years, the most commonly used drugs:

2.3. History of digestive tract disease, if any, please name it specify and list the most commonly used drugs: _______________________________________________________________

2.4. Colorectal surgery / other abdominal surgery history, if any, please specify:

2.5 History of tricyclic antidepressants, if any, please specify:

1. Family history
   1. Family history of colon cancer, if any, please specify:

🞎No 🞎Yes (If yes, please record the following details)

1. History of allergy

🞎No 🞎Yes (If yes, please record the following details)

- 1. Allergic to medication

- 1. Allergic to food

- 1. Contact allergy

1. Habits and customs
   1. Smoking years, tobacco pack /day
   2. Alcohol abuse years, alcohol consumption ML / each time

Note: Non-smoking / drinker for 0 years, no need for later.Non-smoking / drinking over 6 months belong to quitting smoking / abstinence

**Recent defecation condition**

1. Defecation frequency: time / day, or time / week
2. Select Yes if at least one of 4 bowel movements

🞎 defecation laborious 🞎 defecation or obstruction

🞎 defecation is massive or hard stool 🞎 defecation requires manual assistance or laxative

1. Most of the stool characteristics in the last month are:

🞎 Type 1: Separate hard lumps, like nuts (hard to pass)

🞎 Type 2: Sausage-shaped but lumpy

🞎 Type 3: Like a sausage but with cracks on its surface

🞎 Type 4: Like a sausage or snake, smooth and soft

🞎 Type 5: Soft blobs with clear-cut edges (passed easily)

🞎 Type 6: Fluffy pieces with ragged edges, a mushy stool

🞎 Type 7: Watery, no solid pieces ENTIRELY LIQUID

(According to The Bristol Stool Form Scale)

**Adverse reactions and quality score related to bowel preparation**

1. The number of times you underwent a colonoscopy is: No.
2. The main purpose of the colonoscopy is:

🞎 Health screening 🞎 Disease and diagnosis 🞎 Disease follow-up

1. Anesthesia status at this colonoscopy:

🞎 painless colonoscopy 🞎 common colonoscopy

1. Are there the following symptoms before bowel preparation?

(please check the corresponding symptoms.)

🞎 nausea 🞎 vomiting 🞎 abdominal pain 🞎 bloating 🞎 others,

1. Adverse reactions occurring during intestinal preparation:

(please score according to the severity of symptoms , 0 points for no discomfort, 10 points for strong discomfort and intolerance)

| Symptoms | No | Yes | Corresponding scores according to the severity of symptoms |
| --- | --- | --- | --- |
| nausea | 🞎 | 🞎 | points |
| vomiting | 🞎 | 🞎 | points |
| abdominal pain | 🞎 | 🞎 | points |
| bloating | 🞎 | 🞎 | times |
| others | 🞎 | 🞎 | Please specify: (symtoms), points |

1. Does laxative intake 100% of the specified amount: 🞎Yes 🞎No
2. Are there any additional supplementary doses of PEG or other oral laxatives?

🞎 No 🞎 Yes,

1. Total time of taking the laxative medication: a total of minutes

About minutes at 1L; about minutes for 2-3L.

1. Would you like to have another colonoscopy if necessary?

🞎Yes 🞎No

10. Bowel preparation quality score for colonoscopy (assessed by the colonoscopist)

| Each segment of the colon | BBPS score |
| --- | --- |
| Left colon |  |
| Transverse colon |  |
| Right colon |  |
| Total colon (BBPS total score) |  |

Note: the cleanliness of each segment of colon is scored as 0, 1, 2 and 3 in turn, with the highest score of 9 and the lowest score of 0.

**Reports of Adverse Events**

| Is there any adverse event： 🞎No  🞎Yes (If yes, please fill in the form below) | | |
| --- | --- | --- |
| Please record the events in details, including symptoms, signs, time, related test results and correspondent treatment with medical terminology: | | |
| Name/General  description |  |  |
| Severity | 🞎Mild  🞎Moderate  🞎Severe | 🞎Mild  🞎Moderate  🞎Severe |
| Is there any  correspondent  treatment | 🞎No  🞎Yes (If Yes, please tick the correspondent treatment)  Auricular acupressure：  🞎temporarily stop using auricular acupressure   🞎permanent stop using auricular acupressure   🞎not applicable  🞎not known  Using other medications or other therapies：  🞎No  🞎Yes, please record these briefly describe the process and related treatment: | 🞎No  🞎Yes (If Yes, please tick the correspondent treatment)  Auricular acupressure：  🞎temporarily stop using auricular acupressure   🞎permanent stop using auricular acupressure   🞎not applicable  🞎not known  Using other medications or other therapies：  🞎No  🞎Yes, please record these briefly describe the process and related treatment: |
| Correlation to  auricular acupressure | 🞎Certain  🞎Probably/Likely  🞎Possible  🞎Unlikely  🞎Conditional/Unclassified  🞎Unassessable | 🞎Certain  🞎Probably/Likely  🞎Possible  🞎Unlikely  🞎Conditional/Unclassified  🞎Unassessable |
| Outcome | 🞎Death 🞎Symptom continue 🞎Symptom relieved or disappeared  🞎Symptom relieved or disappeared but with sequela   🞎Symptom improving 🞎Not known | 🞎Death 🞎Symptom continue 🞎Symptom relieved or disappeared  🞎Symptom relieved or disappeared but with sequela   🞎Symptom improving 🞎Not known |
| Withdrawal | 🞎No  🞎Yes | 🞎No  🞎Yes |
| Is it a severe adverse  event | 🞎No 🞎Yes (If yes, please tick the correspondent ones below)  🞎 Lethal  🞎Life-threatening   🞎Require hospitalization or prolong hospitalization duration   🞎Lead to apparent or permanent disability or loss of working ability 🞎Lead to other critical medical incident | 🞎No 🞎Yes (If yes, please tick the correspondent ones below)  🞎 Lethal  🞎Life-threatening   🞎Require hospitalization or prolong hospitalization duration   🞎Lead to apparent or permanent disability or loss of working ability 🞎Lead to other critical medical incident |

Notes：

1. Adverse events include abnormal test results that were normal at recruitment. And the changes could not be explained by the development of the condition.

2. Severity of adverse events：Mild: temperate symptom that has no impact on daily living and requires simple treatment or no treatment; moderate: discomfort symptom that has an impact on daily living, but can be relieved with treatment and would not lead to severe or permanent harm patient; severe: seriously impact daily living or the development of spasticity, or requiring

stronger or larger dose of treatment.

3. Please copy and attach the additional pages of “Reports of Adverse Events” if there are more than two adverse events reported.

Signature of investigator: Date:
